# Pharmacologic Treatment of Metabolic Dysfunction-Associated Steatotic Liver Disease in the Context of Type 2 Diabetes

**DOI:** 10.1007/s11892-026-01621-w

**Published:** 2026-03-14

**Authors:** Konstantinos Malandris, Konstantinos Charalampidis, Rohit Loomba, Emmanouil Sinakos

**Affiliations:** 1 Clinical research and evidence-based medicine Unit, Aristotle University of Thessaloniki, Thessaloniki, Greece; 2 Fourth Department of Internal Medicine, Hippokratio Hospital, Aristotle University of Thessaloniki, Thessaloniki, 54642 Greece; 3 MASLD Research Center, Division of Gastroenterology and Hepatology, University of California at San Diego, Altman Clinical and Translational Research Institute, La Jolla, San Diego, CA USA; 4 Medicine and Hepatology, Aristotle University of Thessaloniki, Hippokratio Hospital, 49, Konstantinoupoleos Str, Thessaloniki, 54642 Greece

**Keywords:** MASLD, MASH, T2D, Pharmacological management

## Abstract

**Purpose of review:**

Metabolic dysfunction–associated steatotic liver disease (MASLD) is highly prevalent among individuals with type 2 diabetes (T2D). This review summarizes current evidence on the pharmacologic treatment of MASLD, with emphasis on agents currently approved for the management of T2D.

**Recent findings:**

Among glucose-lowering therapies, GLP-1 RAs, dual GIP/GLP-1 RAs, pioglitazone, and SGLT2 inhibitors demonstrate meaningful benefits in steatohepatitis. Semaglutide provides the most robust evidence for fibrosis benefit, while data suggest potential antifibrotic effects of tirzepatide and dapagliflozin. Resmetirom and semaglutide are the only agents specifically approved for MASLD. An expanding pipeline of dual glucagon/GLP-1 and triple GIP/GLP-1/glucagon agonists such as retatrutide has shown marked reductions in liver fat and signals of MASH benefit. Additional therapies, including pan-PPAR agonists, and FGF21 analogues are advancing through phase 3 development.

**Summary:**

The therapeutic landscape is rapidly evolving toward integrated metabolic, hepatic, and cardiovascular risk reduction in T2D and MASLD.

## Introduction

Metabolic dysfunction-associated steatotic liver disease (MASLD) is defined as excessive hepatic fat accumulation in the presence of specific cardiometabolic risk factors (including type 2 diabetes), after exclusion of secondary causes of liver steatosis [1, 2]. Epidemiological estimates suggest a global prevalence of approximately 38%, making MASLD the most prevalent hepatic disorder worldwide [3]. Its progressive form, metabolic dysfunction–associated steatohepatitis (MASH), is characterized by ballooning and lobular inflammation leading to development of fibrosis and eventually cirrhosis [4]. Projections indicate that by 2040, the prevalence of MASLD may rise to 55% globally, mirroring the increasing prevalence of type 2 diabetes (T2D) and obesity [5].

Given their shared pathophysiological background, MASLD and T2D frequently coexist [6]. Approximately two-thirds of individuals with T2D have MASLD, and up to one-third develops MASH [7]. Fibrosis stage is the principal determinant of MASLD prognosis, with advanced fibrosis strongly associated with increased all-cause and liver-related mortality, while accumulating evidence indicates that T2D independently promotes fibrosis progression [8, 9]. Furthermore, among people with biopsy-confirmed MASH and compensated cirrhosis, T2D is associated with a four-fold increase in mortality and nearly a two-fold increase in liver-related events, over a median follow-up of five years [1, 10].

As with T2D, MASLD is now recognized as a significant and independent risk factor for adverse cardiovascular events, which represent the leading cause of death in this population [11]. In a cohort study of 10,422 middle-aged individuals with histologically confirmed MASLD and nearly 50,000 matched controls, MASLD was associated with an increased risk of adverse cardiovascular events over a median follow up of 13.6 years [12]. This risk was independent of common cardiometabolic risk factors and increased progressively with worsening severity of MASLD histology. These findings are supported by a recent meta-analysis of more than 5.5 million participants, that reported a 45% higher risk of cardiovascular events in individuals with MASLD [13]. This excess risk increased further with advancing fibrosis stage and was independent of age, sex, adiposity indices, and diabetes status.

Given the close interplay between MASLD and T2D, and in light of recent advances in the therapeutic landscape of MASLD, this review aims to summarize evidence for the pharmacological management of MASLD, with a particular focus on agents currently approved for the treatment of T2D in order to assist diabetologists in their choice of treatment in everyday clinical practice.

## Glucose-Lowering Drugs Approved for T2D and Effect on MASLD

When managing individuals with T2D, healthcare providers traditionally prioritize two goals: achieving and maintaining glycemic targets, and reducing cardiovascular and kidney risk in high-risk individuals [14]. Given the increased cardiovascular risk associated with MASLD, together with the close and bidirectional relationship between MASLD, T2D, and other cardiometabolic risk factors, the selection of glucose-lowering therapies should also take into account their potential to provide additional liver-related benefits, including improvements in histological parameters.

### Metformin

Metformin is commonly used as the first-line agent for the management of hyperglycemia in individuals with T2D. Through its insulin-sensitizing effects, metformin typically reduces HbA1c by approximately 1% and leads to a modest weight reduction [15, 16], while having a neutral effect on major adverse cardiovascular outcomes [2]. Metformin does not provide any meaningful histological improvement in MASLD and should not be used for this purpose [1, 2, 17]. Although observational studies have suggested reduced mortality and a lower incidence of hepatocellular carcinoma (HCC) among people with T2D and MASH-related bridging fibrosis or compensated cirrhosis [18], a recent meta-analysis that accounted for statin and aspirin use did not demonstrate a clear protective effect of metformin on HCC incidence [19].

### Dipeptidyl Peptidase-4 Inhibitors

Dipeptidyl peptidase-4 inhibitors (DPP4is) are commonly used on top of metformin for the management of hyperglycemia especially in cases of severe renal impairment. Owing to their low risk of hypoglycemia, DPP4is are also suitable for frail individuals in whom a less stringent glycemic target is appropriate. By enhancing the action of endogenous incretin hormones, DPP4is lower HbA1c by approximately 0.6% [15]. They do not promote weight loss and have not demonstrated benefit on major adverse cardiovascular outcomes [2, 16]. Histological data regarding the efficacy of DPP4is in people with MASLD are limited [2]. In a small randomized controlled trial (RCT), 12 participants with T2D and histologically confirmed MASH were allocated to either sitagliptin 100 mg/day or placebo [20]. After 24 weeks, sitagliptin did not improve liver fibrosis or MASH activity. In another RCT, 50 participants with MASLD and either prediabetes or T2D were allocated to sitagliptin 100 mg/day or placebo for 24 weeks [21]. In this case, MASLD was defined based on magnetic resonance imaging proton density fat fraction (MRI-PDFF) and fibrosis was assessed with magnetic resonance elastography. At study completion, no significant differences were observed between treatment groups in either MRI-PDFF or MRE. As such, DPP4is should not be considered for the management of MASLD in people with T2D [2, 22]. With the exception of vildagliptin, this drug class can be used in patients with cirrhosis and moderately impaired liver function, but are not recommended in cases of severe hepatic impairment [23].

### Sulfonylureas

Despite their ability to lower HbA1c by roughly 1%, the use of sulphonylureas in people with T2D has progressively declined mainly due to their increased risk of hypoglycemia and the absence of renal, cardiovascular, or weight-reduction benefits compared with existing medications [15]. To date, there are no RCTs with histological endpoints assessing the efficacy of this drug class in MASLD [22, 24]. Results of RCTs in people with T2D and MASLD have reported minor benefits in liver steatosis based on noninvasive indices [25]. Given the increased risk of hypoglycemia related to sulphonylureas use especially in individuals with cirrhosis and impaired liver function, and the lack of evidence on histological endpoints sulphonylureas should not be considered for the management of MASLD [1, 23].

### Insulin

Insulin initiation should be considered regardless of background treatment in people with markedly elevated HbA1c levels or severe hyperglycemia [14]. Moreover, insulin is the preferred pharmacologic option for managing hyperglycemia in decompensated cirrhosis [26]. Despite its propensity to cause weight gain, the increased risk of hypoglycemia, and its neutral effect on cardiovascular and renal outcomes [2], results from RCTs in participants with MASLD and T2D have reported a beneficial effect on liver steatosis as assessed by means of MRI-PDFF with a relative reduction from baseline ranging from 5% to 11% [27, 28]. Whether these improvements in steatosis translate into favourable effects on MASH activity or fibrosis remains unclear [29]. Consequently, insulin should not be used for the treatment of MASLD in people with T2D. Nevertheless, clinicians should remain aware of its antisteatotic effect despite its weight-promoting properties.

### Thiazolidinediones (Pioglitazone)

Pioglitazone is a peroxisome proliferator-activated receptor (PPAR)-γ agonist that enhances insulin sensitivity and promotes triglyceride storage in adipose tissue, thereby reducing ectopic lipid deposition in the liver [22, 29]. In the first RCT, 55 individuals with prediabetes or T2D and histological confirmed MASH were allocated to pioglitazone 45 mg/daily or placebo on top of a hypocaloric diet [30]. After six months of treatment, pioglitazone significantly improved all components of NAS score [steatosis (65% vs. 38%, *p* = 0.03), ballooning (54% vs. 24%, *p* = 0.02), and lobular inflammation (65% vs. 29%, *p* = 0.008)] compared with placebo, although changes in fibrosis did not differ significantly between groups (46% vs. 33%, *p* = 0.08). In another study of longer duration (18 months), 101 participants with prediabetes or T2D and histological confirmed MASH were randomly allocated to either pioglitazone 45 mg/daily or placebo [31]. Pioglitazone resulted in a greater proportion of participants achieving a ≥ 2-point reduction in NAS without worsening of fibrosis (58% vs. 17%, *p* < 0.001) and a higher rate of MASH resolution (51% vs. 19%, *p* < 0.001). However, there was no significant difference in the proportion of patients achieving ≥ 1-point improvement in fibrosis between groups (20% vs. 13%, *p* = 0.13). More recently, an RCT with paired biopsy data randomized 105 individuals with T2D and MASH in a 1:1:1 ratio to placebo, vitamin E, or combination therapy with vitamin E plus pioglitazone 45 mg/daily [32]. Combination therapy significantly increased the proportion of participants achieving a ≥ 2-point reduction in NAS without worsening of fibrosis (54% vs. 19%, *p* = 0.003), whereas vitamin E alone did not (31% vs. 19%, *p* = 0.26). Neither active treatments showed a clear fibrosis benefit. The beneficial effect of pioglitazone on MASH resolution appears consistent in RCTs involving people without diabetes as well [33–35]. Across trials, improvements in fibrosis have been modest, with placebo-subtracted fibrosis response rates typically ranging from 9% to 22% [2]. It is important to note that most studies were underpowered for fibrosis endpoints. Meta-analyses confirm the beneficial effect of pioglitazone on MASH resolution, while also providing evidence for fibrosis improvement [36, 37]. As a result, pioglitazone should be considered for the management of MASH in people with T2D. Nevertheless, it should be noted that its primary benefit lies in improving hepatic inflammation, while evidence supporting its antifibrotic effects is comparatively weaker. Moreover, the use of pioglitazone requires careful consideration due to concerns about fluid retention and potential worsening of heart failure, as well as its limited applicability in individuals with decompensated cirrhosis [14, 23].

### Glucagon-Like peptide-1 Receptor Agonists (GLP-1 RAs)

Since their introduction, GLP-1 RAs have emerged as a leading drug class for the management of T2D. This is driven by their robust glucose-lowering efficacy [15, 38], clinically meaningful weight loss [38], and demonstrated protective effects against major adverse cardiovascular and renal events [39].

Among the various GLP-1 receptor agonists, liraglutide was the first to demonstrate therapeutic potential in the management of MASH. In a small phase 2 trial, 52 individuals with biopsy-confirmed MASH (33% with T2D) were randomized to liraglutide 1.8 mg/daily or placebo for 48 weeks [40]. Treatment with liraglutide resulted in MASH resolution in 39% of participants compared with 9% in the placebo group (relative risk 4.3, 95% CI 1.0–17.7). Furthermore, participants in the liraglutide group experienced a lower rate of fibrosis progression compared with placebo.

Semaglutide is the GLP-1 RA with the largest amount of data on MASH histological endpoints. In a phase 2 RCTs of paired histological data, 320 patients with non-cirrhotic MASH (62% with T2D) were allocated to semaglutide 0.1 mg/daily or 0.2 mg/daily or 0.4 mg/daily or placebo for 72 weeks [41]. At the end of treatment, MASH resolution without worsening of fibrosis was achieved in 40% of participants in the 0.1-mg group, 36% in the 0.2-mg group, 59% in the 0.4-mg group, and 17% in the placebo group (*P* < 0.001 for semaglutide 0.4 mg vs. placebo). Fibrosis improvement without worsening of MASH occurred in 43% of participants in the 0.4-mg group and in 33% in the placebo group (*P* = 0.48). Results were similar in subgroup analyses based on diabetes status. Recently, the FDA approved semaglutide 2.4 mg/weekly for the management of non-cirrhotic MASH following the results of a pre-planned interim analysis of the phase 3 ESSENCE trial [42]. In this study 800 participants (56% with T2D) were randomly allocated to either semaglutide 2.4 mg/weekly or placebo. After 72 weeks, 62.9% of the 534 participants in the semaglutide group achieved resolution of MASH without worsening of fibrosis compared with 34.3% of the 266 patients in the placebo group (estimated difference, 28.7%, 95% CI, 21.1 to 36.2; *p* < 0.001). Similarly, fibrosis improvement without worsening of MASH was reported in 36.8% in the semaglutide group and in 22.4% in the placebo group (estimated difference, 14.4%, 95% CI, 7.5 to 21.3; *p* < 0.001) [43]. Of note, the mean change form baseline in body weight was considerably greater in the semaglutide group compared with placebo. Nevertheless, mediation analyses suggest that both weight loss–dependent and weight loss–independent metabolic mechanisms contribute to semaglutide-induced improvements in histological endpoints [44]. Similarly, post hoc subgroup analyses demonstrated that the proportion of participants achieving either MASH resolution or fibrosis improvement was consistently higher with semaglutide than with placebo, irrespective of glycemic status [45]. A phase 2 RCT with paired histological assessments evaluated the efficacy of semaglutide in 71 patients with MASH-related compensated cirrhosis (75% with T2D) [46]. After 48 weeks, there was no difference between semaglutide 2.4 mg/weekly and placebo in fibrosis improvement (odds ratio 0.28, 95% CI 0.06–1.24; *p* = 0.087). Nonetheless, the overall incidence of adverse events was similar between groups (89% vs. 79%), and no worsening of hepatic function or decompensating events attributable to semaglutide were observed. More recently, the results of the SAMARA trial have demonstrated that non-invasive tests can reliably identify patients with at-risk MASH who are eligible for treatment with semaglutide and can also be used to monitor therapeutic response, obviating the need for liver biopsy assessment [47].

Histological data for other GLP-1 receptor agonists are currently lacking. However, evidence indicates that dulaglutide 1.5 mg weekly and exenatide 10 mcg twice daily can reduce liver steatosis by approximately 32% and 41%, respectively, as assessed by MRI-PDFF in people with T2D and MASLD [48, 49]. This is clinically relevant, as a ≥ 30% relative reduction in MRI-PDFF is associated with higher odds of histological improvement and MASH resolution [50].

Consequently, among available GLP-1 RAs, semaglutide and liraglutide have proven efficacy in MASH resolution. To date, only semaglutide has shown a favorable effect on fibrosis however, this antifibrotic benefit appears to occur only at the obesity-approved dose of 2.4 mg weekly. Other GLP-1 RAs, such as dulaglutide, may also lead to MASH resolution, although this conclusion is inferred indirectly from non-invasive assessments.

### Dual Glucose-Dependent Insulinotropic Polypeptide (GIP) / GLP1-Receptor Agonists (tirzepatide)

Tirzepatide is a novel dual GIP/GLP-1 RA approved for the management of T2D and obesity. Meta-analyses demonstrate its superiority over semaglutide with respect to both HbA1c reduction and weight loss [51]. Furthermore, recently published data indicate that tirzepatide is noninferior to dulaglutide for the composite endpoint of cardiovascular death, myocardial infarction, or stroke in individuals with T2D and established atherosclerotic cardiovascular disease [52].

In an MRI-PDFF substudy of the SURPASS-3 trial, tirzepatide 10 mg and 15 mg achieved reductions in liver fat content of 47% and 39%, respectively, compared with insulin degludec in individuals with T2D [28]. Further insights come from a phase 2 RCT with paired liver histology, in which 190 individuals with non-cirrhotic MASH (58% with T2D) were randomized to tirzepatide 5 mg/weekly, 10 mg/weekly, or 15 mg/weekly, or placebo, for 52 weeks [53]. At study end, MASH resolution without worsening of fibrosis occurred in 10% of participants receiving placebo, compared with 44%, 56%, and 62% in the 5-mg, 10-mg, and 15-mg tirzepatide groups, respectively (*p* < 0.001 for all tirzepatide doses vs. placebo). Fibrosis improvement without worsening of MASH was achieved in 30% of the placebo group, versus 55% with tirzepatide 5 mg (estimated difference 25%; 95% CI, 5–46), 51% with tirzepatide 10 mg (estimated difference 22%; 95% CI, 1–42), and 51% with tirzepatide 15 mg (estimated difference 21%; 95% CI, 1–42). Results from participant level exploratory analyses for the same trial suggest that in the T2D subgroup, participants who achieved either MASH resolution or fibrosis improvement (responders) had greater weight and HBA1c reduction compared to non-responders. Similar to semaglutide, mediation analyses revealed that both weight loss–dependent and weight loss–independent mechanisms contribute to tirzepatide-induced improvements in histological endpoints [54].

As a result, tirzepatide should be considered for the management of MASH in people with T2D. Nevertheless, its primary benefit appears to be the improvement of hepatic inflammation, while evidence supporting antifibrotic effects is comparatively weaker. These estimates derive from a single RCT and require confirmation in future trials of longer duration.

### Sodium-Glucose Co-Transporter 2 (SGLT2) Inhibitors

SGLT2 inhibitors lower blood glucose by inhibiting renal glucose reabsorption, thereby increasing urinary glucose excretion [22]. In addition to their moderate-to-high glucose-lowering efficacy, these agents confer substantial cardiovascular and renal benefits in individuals with T2D [55], while also promoting weight loss [16].

Evidence for the efficacy of SGLT2 inhibitors in individuals with MASLD and T2D largely derives from RCTs using non-invasive biomarkers, mainly MRI-PDFF [56]. Across these studies, SGLT2 inhibitors have consistently demonstrated improvements in hepatic steatosis, with reductions in MRI-PDFF ranging from 10% to 31%, with empagliflozin generally producing the largest effects. More recently, an RCT evaluated dapagliflozin 10 mg/daily in individuals with MASH using paired histological data [57]. After 48 weeks of treatment, MASH resolution without worsening of fibrosis occurred in 23% of participants receiving dapagliflozin versus 8% in the placebo group (*p* = 0.01). Similarly, fibrosis improvement without worsening of MASH was observed in 45% of the dapagliflozin group compared with 20% of the placebo group (*p* = 0.001).

**Fig. 1:**
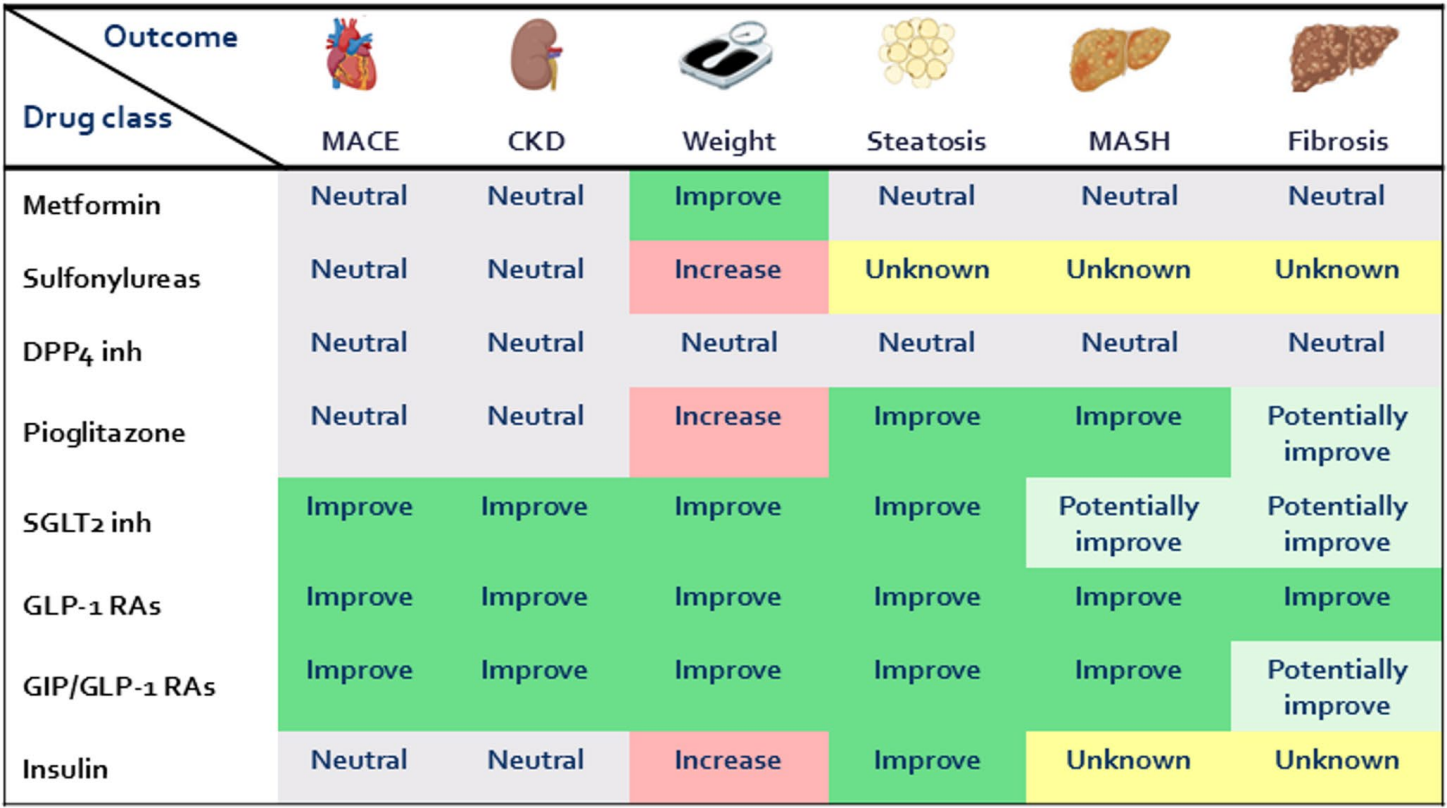
Effects of glucose-lowering drugs approved for T2D in MASLD CKD; Chronic kidney disease, DPP4; Dipeptidyl peptidase-4, GIP; Glucose-dependent insulinotropic polypeptide, GLP-1; Glucagon like peptide 1, Inh; Inhibitor, MACE; Major adverse cardiovascular events, MASH; Metabolic dysfunction associated steatohepatitis, MASLD; Metabolic dysfunction associated steatotic liver disease, RAs; Receptor agonists, SGLT2; Sodium-Glucose Co-transporter 2

Overall, SGLT2 inhibitors are able to improve steatosis while, indirect evidence form noninvasive indices suggest their potential to improve steatohepatitis. To date, only dapagliflozin has demonstrated favourable effects on both hepatic inflammation and fibrosis histologically however, these findings require further confirmation in future trials. Evidence regarding the effect of this drug class in individuals with cirrhosis remains limited. In a population-based cohort study, SGLT2 inhibitors were associated with a lower risk of hepatic decompensation compared with thiazolidinediones, while being comparable to GLP-1 RAs [58]. Figure 1 summarizes the existing evidence on the effect of all the approved glucose-lowering drugs on weight, renal and cardiovascular outcomes as well as MASLD-related outcomes.

## Approved and Emerging Therapies Solely for the management of MASLD

### Approved Drugs

As of December 2025, the FDA had approved semaglutide 2.4 mg/weekly and resmetirom -a thyroid hormone receptor-β (THR-β) agonist- 80/100 mg daily for the management of non-cirrhotic MASH. EMA has also approved resmetirom for the same indication. The evidence of semaglutide effectiveness in MASLD is presented in the previous section of this review.

Resmetirom’s regulatory approval was based on the positive results of the phase 3 MAESTRO-NASH trial [59]. In this study, 966 participants with non-cirrhotic MASH (67% with T2D) were randomized to receive oral resmetirom 80 mg/daily, 100 mg/daily, or placebo. After 52 weeks of treatment, both resmetirom doses achieved higher rates of MASH resolution without worsening of fibrosis—25.9% with 80 mg and 29.9% with 100 mg—compared with 9.7% in the placebo group. Similarly, fibrosis improvement without worsening of MASH occurred more frequently with resmetirom (24.2% and 25.9% with the two doses) than with placebo (14.2%). These findings were consistent in subgroup analyses of participants with T2D. Notably, resmetirom therapy produced significant reductions in low-density lipoprotein cholesterol of up to 16%, with no apparent effect on body weight.

Given that cardiovascular mortality constitutes the leading cause of death in individuals with MASH, the cardiovascular profile of approved therapies is of critical clinical importance. However, there remains a paucity of evidence regarding the effect of resmetirom on cardiovascular outcomes, as dedicated trials have not yet been conducted. In contrast, semaglutide is supported by large, well-conducted cardiovascular outcomes trials demonstrating significant cardiovascular benefits in individuals with T2D, as well as in people with overweight or obesity without diabetes.

### Emerging Therapies

Despite several unsuccessful candidates in previous years, many agents have already shown promising results in phase 2 trials of histological endpoints and are currently investigated in phase 3 trials. The evidence for these drugs is presented below with an emphasis on their possible effect on T2D. They target either metabolism or liver-specific pathways of hepatocyte injury, including fatty acid uptake, de novo lipogenesis, triglyceride formation, oxidative stress, inflammation and cell death [60]. In addition, glucose-lowering medications (Table 1) and other emerging agents with distinct mechanisms of action (Table 2) are under investigation for the management of MASH, either as monotherapy or in combination regimens.

**Table 1:** Notable ongoing studies of glucose-lowering agents on MASH and T2D population

Study Identifier (Study phase)	Drug Name	Drug class	Population	Duration	Dosage	Administration method	Primary Endpoint	Status
COMBATT2NASH NCT04639414 (Phase IV)	Empagliflozin + semaglutide vs. empagliflozin monotherapy or placebo	SGLT2 inh + GLP-1RAs	MASH and T2D (fibrosis stage F1–F3)	48 weeks	Empagliflozin 10 mg daily, Semaglutide 1 mg weekly	Once daily tablet and once weekly subcutaneous injections	Resolution of MASH without worsening of fibrosis	Active, not recruiting
UMIN000045003	Luseogliflozin + semaglutide vs. semaglutide monotherapy	SGLT2 inh + GLP-1RAs	MASH and T2D (Japanese cohort)	52 weeks	Luseogliflozin 2.5 mg daily, Semaglutide 0.25 mg to 0.5 mg weekly	Once daily tablet and once weekly subcutaneous injections	Resolution of MASH without worsening of fibrosis	Active, not recruiting

*GLP-1 *Glucagon like peptide 1,* Inh *Inhibitor,* MASH *Metabolic dysfunction associated steatohepatitis,* RAs *Receptor agonists, *SGLT2 *Sodium-Glucose Co-transporter 2,* T2D *Type 2 diabetes

**Table 2:** Notable ongoing phase 2 studies on MASH population

Study Identifier (Study phase)	Drug Name	Drug class	Population	Duration	Dosage	Administration method	Primary Endpoint	Status
NCT06937749 (Phase 2)	Mazdutide	GLP-1 and glucagon dual RA	MASH F2 or F3 fibrosis	60 weeks	Not available	Weekly subcutaneous injection	Resolution of MASH without worsening of fibrosis	Recruiting
MK-6024–013 NCT05877547 (Phase 2b)	Efinopegdutide	GLP-1 and glucagon dual RA	MASH F2 or F3 fibrosis	52 weeks	4 mg, 7 mg, 10 mg	Weekly subcutaneous injection	Resolution of MASH without worsening of fibrosis	Active, not recruiting
Horizon NCT05583344 (Phase 2b)	GSK4532990	17 β-hydroxysteroid dehydrogenase type 13 minimization	MASH F3 or F4 fibrosis	52 weeks	100 mg (monthly), 200 mg (three monthly)	Monthly subcutaneous injections	≥ 1 Stage improvement in fibrosis without worsening of MASH - F3 Cohort	Active, not recruiting
NCT05692492 (Phase 2b)	ZSP1601	Pan-phosphodiesterase inh	MASH F2 or F3 fibrosis	48 weeks	50 mg, 100 mg	Twice daily oral tablet	Improvement of MASH without worsening of fibrosis OR ≥ 1 stage improvement in fibrosis without worsening of MASH	Active, not recruiting
NCT04697810 (Phase 2b)	Namodenoson	A3 adenosine RA	MASH F2 or F3 fibrosis	36 weeks	25 mg	Twice daily oral tablet	≥ 2-point improvement in NAS	Recruiting
MONARCH NCT06108219 (Phase 2b)	Miricorilant	Selective glucocorticoid receptor modulator	MASH F2 or F3 fibrosis	48 weeks	100 mg, 200 mg	Twice weekly oral tablet	Change from baseline in liver-fat content assessed by MRI-PDFF	Recruiting
FORTUNA NCT05809934 (Phase 2b)	AZD2693	PNPLA3 antisense oligonucleotide	MASH F2 or F3 fibrosis plus carriers for the PNPLA3 rs738409 148 M risk allele	52 weeks	Not available	Weekly subcutaneous injection	MASH resolution without worsening of fibrosis	Active, not recruiting
CENTRICITY NCT05623189 (Phase 2b)	HTD1801	AMP-activated protein kinase activator combined secondary bile acid	MASH F2 or F3 fibrosis plus T2D or prediabetes	60 weeks	1250 mg	Twice daily oral tablet	≥ 2-point improvement in NAS without worsening of fibrosis OR resolution of MASH	Active, not recruiting
NCT05011305 (Phase 2b)	Saroglitazar magnesium	Dual PPAR-α/γ agonist	MASH F2 or F3 fibrosis	52 weeks	2 mg, 4 mg	Once daily oral tablet	Resolution of MASH without worsening of fibrosis	Active, not recruiting
NCT05016882 (Phase 2)	Zalfermin and semaglutide	FGF21 + GLP-1 RA	MASH F2, F3, and F4 fibrosis	19 months	NNC0194–0499 7·5 mg plus semaglutide 2·4 mg	Weekly subcutaneous injection	Improvement in fibrosis without worsening of MASH	Active, not recruiting
NCT05327127 (Phase 2)	K-877-ER (fibrate) and tofogliflozin	Fibrate + SGLT2 inh	MASH F1 to F3 fibrosis	48 weeks	Not available	Daily oral tablets	Improvement in MASH without worsening of fibrosis	Recruiting

*FGF *Fibroblast growth factor,* GLP-1 *Glucagon like peptide 1,* Inh *Inhibitor,* MASH *Metabolic dysfunction associated steatohepatitis,* MRI-PDFF *Magnetic resonance imaging proton density fat fraction,* NAS *Non-alcoholic fatty liver disease (NAFLD) activity score,* PPAR *Peroxisome proliferator activated receptor,* RAs *Receptor agonists,* SGLT2 *Sodium-Glucose Co-transporter 2,* T2D *Type 2 diabetes

### Incretin-Based Polyagonists

Survodutide is a novel dual glucagon/GLP-1 RA administered subcutaneously. In a phase 2 RCT, 293 participants with non-cirrhotic MASH (39% with T2D) were assigned to three different weekly doses of survodutide (2.4 mg, 4.8 mg, or 6.0 mg) or placebo [61]. After 48 weeks of treatment, survodutide achieved higher rates of MASH improvement without worsening of fibrosis (47%, 62%, and 43% in the 2.4 mg, 4.8 mg, and 6.0 mg groups, respectively compared with 22% in the placebo group). Fibrosis improvement rates were numerically higher with survodutide (34%, 36%, and 34% across the three doses) versus placebo (22%). Subgroup analyses based on diabetes status are not currently available. However, in a recently published placebo- and active-controlled trial in individuals with T2D, survodutide produced greater reductions in HbA1c and body weight compared with both placebo and semaglutide 1 mg [62]. Notably, high-dose survodutide led to ≥ 5% weight loss in more than 50% of participants and ≥ 10% weight loss in more than 25% [62]. Survodutide will be further evaluated in the phase 3 LIVERAGE trial (NCT06632444), which will enroll participants with MASH and F2–F3 fibrosis. Mazdutide, another once-weekly dual glucagon/GLP-1 receptor agonist, achieved resolution of steatosis in more than 70% of participants in a recently announced 48-week analysis of a clinical trial conducted in individuals with obesity [63]. This agent has also shown promising glucose-lowering effects in individuals with T2D, achieving greater reductions in HbA1c compared with semaglutide (-2.03% vs. -1.84%) [64]. In a recent phase 2 RCT of pemvidutide, MASH resolution without worsening of fibrosis was noted in 58% and 52% (two different dose regiments) compared to 20% in the placebo group at 24 weeks with no significant difference in fibrosis improvement without worsening of MASH [63]. Retatrutide is a first-in-class, once-weekly triple agonist targeting GIP, GLP-1, and glucagon receptors. In a phase 2 RCT including 281 participants with T2D, retatrutide administered at 12 mg once-weekly, achieved superior reduction in HbA1c (up to 2.02%) at 24 weeks, significantly outperforming placebo and active comparator dulaglutide (1.41%) [65]. In another placebo-controlled phase 2 trial of obese, non-diabetic participants retatrutide achieved a mean weight loss of 24.2% at 48 weeks [66]. A prespecified substudy of this obesity trial assessed hepatic outcomes in participants with MASLD and baseline liver fat content ≥ 10% measured by MRI-PDFF [67]. By week 48, participants receiving the highest doses (8 mg and 12 mg) achieved a mean relative liver fat reduction of 81–86%. Resolution of steatosis, defined as liver fat content < 5%, was observed in more than 90% of participants treated with the 12 mg dose. A dedicated phase 3 RCT evaluating the cardiovascular safety and efficacy of retatrutide is currently ongoing (NCT06383390), with the aim of assessing its impact on major adverse cardiovascular outcomes. In parallel, the phase 3 SYNERGY-OUTCOMES trial (NCT07165028) is actively recruiting individuals with high-risk MASLD, identified using noninvasive indices. This large-scale study plans to enroll approximately 4,500 participants and is designed to compare retatrutide, tirzepatide, and placebo with respect to major adverse liver outcomes. Lastly, in the ongoing DD01-DN-02 (NCT06410924) phase 2 study, DD01, another once-weekly dual glucagon/GLP-1 RA met the primary endpoint at week 12 in reducing liver fat by more than 30% in 75.8% of participants in the intervention group (compared to 11.8% in the placebo group) with liver fat normalization observed in 48.5% [63].

### Fibroblast Growth Factor 21 Analogues

Fibroblast growth factor 21 (FGF21) is a peptide hormone secreted primarily by the liver and, to a lesser extent, by adipose tissue, exerting a broad range of metabolic effects [68, 69]. Efruxifermin, a subcutaneous, long-acting IgG Fc-FGF21 fusion protein, pegozafermin, a subcutaneous, long-acting glycopegylated recombinant FGF21 analogue and efimosfermin alpha, a very long-acting IgG1 Fc-conjugated analogue, are currently studied in phase 3 trials after successful phase 2 results [70].

A phase 2b RCT of 128 participants with biopsy-confirmed MASH with F2 or F3 fibrosis compared two different weekly subcutaneous doses of efruxifermin (28 mg or 50 mg) with placebo [71]. After 96 weeks of treatment, only high dose efruxifermin resulted in significantly higher rates of fibrosis improvement without worsening of MASH compared with placebo (estimated difference 31%; 95% CI, 12 to 42; *p* = 0.003). This favorable effect on fibrosis was more pronounced in individual with T2D and F3 fibrosis. Both efruxifermin doses resulted in higher rates of MASH resolution without worsening of fibrosis—37% to 40%—versus 19% in the placebo group. Although efruxifermin had no major effect on glycemic control (HbA1c increase of 0.2% for efruxifermin 50 mg), there were favorable effects on insulin resistance and lipid profile. In addition, results from a phase 2 trial in individuals with T2D and F1–F3 MASH, which evaluated efruxifermin as an add-on to stable GLP-1 RA therapy, suggest that efruxifermin may serve as a safe adjunctive option to enhance MASLD management in people with diabetes on background antihyperglycemic medication [72]. Efruxifermin is currently being investigated in the phase 3 SYNCHRONY Histology trial (NCT06215716), enrolling participants with MASH and F2–F3 fibrosis.

In a 24-week, phase 2b RCT of 222 individuals with biopsy-proven MASH and F2–F3 fibrosis, pegozafermin administered in three subcutaneous dosing regimens (15 mg and 30 mg once weekly, and 44 mg once every two weeks) was compared with placebo [73]. Both the 30 mg dose (difference vs. placebo 19%; 95% CI, 5–32; *p* = 0.009) and the 44 mg dose (difference vs. placebo 20%; 95% CI, 5–35; *p* = 0.0018) resulted in higher rates of fibrosis improvement without worsening of MASH compared with placebo. In participants with T2D, only the 44 mg regimen retained its antifibrotic effect. All pegozafermin doses produced higher rates of MASH resolution without worsening of fibrosis (37% with 15 mg, 23% with 30 mg, and 26% with 44 mg compared with 2% in the placebo group). In participants with T2D, this effect was retained only with the 30 mg and 44 mg dosing regimens. Although pegozafermin had no meaningful effect on HbA1c (reductions of up to 0.3%) and body weight (reductions of up to 1.2 kg), results suggest a favourable effect on triglyceride levels. The two higher-dose regimens are now being investigated in the phase 3 ENLIGHTEN-fibrosis trial (NCT06318169) in people with MASH and F2–F3 fibrosis.

Efimosfermin alfa is a long-acting FGF21 analogue that, unlike previous agents in this drug class, is administered once monthly. In a 24-week phase 2 trial, monthly efimosfermin 300 mg resulted in higher rates of MASH resolution without worsening of fibrosis, and higher rates of fibrosis improvement without worsening of MASH (68% and 45%, respectively) compared with placebo (29% and 21%, respectively) [74, 75]. The phase 3 ZENITH-1 trial (NCT07221227) is currently ongoing, recruiting participants with MASH and F2–F3 fibrosis.

### Other Agents

Lanifibranor is an orally administered pan-PPAR agonist. In a 24-week, phase 2b RCT, 247 participants with non-cirrhotic MASH (42% with T2D) were allocated to lanifibranor 800 mg/daily, 1200 mg/daily, or placebo [76]. At the end of treatment, both lanifibranor doses achieved higher rates of MASH resolution without worsening of fibrosis (39% with 800 mg and 49% with 1200 mg) compared with placebo (22%). In addition, the 1200-mg dose led to fibrosis improvement without worsening of MASH in 48% of participants, versus 22% in the placebo arm. In participants with MASH and T2D, lanifibranor 1200 mg was superior to placebo for both histological endpoints, with MASH resolution without fibrosis worsening occurring in 45.4% versus 19.5%, and fibrosis improvement without MASH worsening in 51.3% versus 28.8%. Notably, lanifibranor resulted in significant improvements in insulin resistance, with favourable effects on glycemic parameters and lipid profile, despite its associated weight-gaining effects [76, 77]. Lanifibranor is currently under investigation in the ongoing phase 3 NATIV3 trial (NCT04849728), enrolling individuals with biopsy-proven MASH and F2–F3 fibrosis.

ION224 is a novel antisense oligonucleotide DGAT-2 inhibitor, that suppresses de novo lipogenesis. In a recently published phase 2 RCT of 160 participants with MASH (51% with T2D), once-monthly subcutaneous administration of ION224 at doses of 90 mg and 120 mg resulted in significantly higher rates of a ≥ 2-point reduction in NAS and histological resolution of MASH compared with placebo [78].

## Conclusions

Non-pharmacological interventions form the foundation of MASLD management and should be implemented in all individuals irrespective of diabetes status. A well-balanced diet consistent with the Mediterranean dietary principles, combined with regular physical activity tailored to each patient’s needs, is strongly recommended. Weight loss is strongly associated with histological improvement in MASLD, with reductions of approximately 5%, 7%, and 10% linked to improvements in steatosis, MASH activity, and fibrosis, respectively.

Among pharmacologic interventions approved for the management of T2D, GLP-1 RAs, dual GIP/GLP-1 RAs, pioglitazone and SGLT2 inhibitors are able to improve MASH. Semaglutide has the most robust evidence supporting its efficacy in improving liver fibrosis and is now FDA approved for the treatment of MASH in the dose of 2.4 mg weekly. Notably, its beneficial effects on liver histology appear to arise through both weight-dependent and weight-independent mechanisms. Emerging, albeit more limited, evidence also suggests potential antifibrotic effects of dapagliflozin and tirzepatide. Meta-analyses indicate that pioglitazone may confer improvements in fibrosis as well; however, its propensity to cause weight gain and its weaker cardiovascular and renal benefits compared with GLP-1 RAs and tirzepatide make it a less compelling therapeutic choice for many patients. Resmetirom is currently the only agent solely approved for the management of MASLD. Although it has no meaningful effect on body weight, its favorable effects on lipid parameters may translate into additional cardiometabolic benefit in individuals with T2D; however, dedicated cardiovascular outcomes data are currently lacking.

Dual glucagon/GLP-1 receptor agonists and triple GIP/GLP-1/glucagon agonists (notably retatrutide) produce large reductions in liver fat and early signals of MASH benefit, raising the prospect that these agents could become central to MASLD management. Critically, the field is transitioning toward hard clinical endpoints: the phase 3 SYNERGY-Outcomes trial is evaluating whether retatrutide and tirzepatide reduce major adverse liver outcomes in high-risk MASLD, complementing ongoing cardiovascular outcomes programs.

Several additional agents are advancing through phase 3 development following promising phase 2 histologic results. Among them, PPAR panagonist lanifibranor and FGF21 analogues have the highest impact on fibrosis, potentially making them agents of choice in patients with more advanced stages of chronic liver disease. Combinations of FGF21 analogues with more established treatments appear to be safe, although more data on efficacy and safety is needed. It is worth noting that many novel therapies, with different mechanisms of action, are undergoing phase 2 trials with the potential to further expand our therapeutic options.

As more long-term data are emerging and novel therapies are being developed and studied, it should be highlighted that the combination of awareness, early patient identification, lifestyle modifications and use of different drug therapies confer the highest benefit for patients with MASLD and T2D. These optimistic findings should be enhanced by robust data of phase 3 and real-world studies to implement clinical practice guidelines tailored to patients’ needs, values and preferences.

### Human/Animal Studies informed consent statement

This article does not contain any studies with human or animal subjects performed by any of the authors.

## Data Availability

No datasets were generated or analysed during the current study.

## Key References

- Sanyal AJ, Newsome PN, Kliers I, Østergaard LH, Long MT, Kjær MS, et al. Phase 3 Trial of Semaglutide in Metabolic Dysfunction-Associated Steatohepatitis. N Engl J Med. 2025;392:2089-99.
  - ○ A phase 3 RCT providing robust evidence that Semaglutide 2.4 mg weekly can produce both resolution of steatohepatitis and improvement of fibrosis in individuals with MASH and firbosis stage 2 or 3.
- Loomba R, Hartman ML, Lawitz EJ, Vuppalanchi R, Boursier J, Bugianesi E, et al. Tirzepatide for Metabolic Dysfunction-Associated Steatohepatitis with Liver Fibrosis. N Engl J Med. 2024;391:299–310.
  - ○ The first RCT providing robust evidence that Tirzepatide can lead to MASH resoltuion without worsening of fibrosis in individuals with biopsy-confirmed MASH and fibrosis stage 2 or 3.
- Lin J, Huang Y, Xu B, Gu X, Huang J, Sun J, et al. Effect of dapagliflozin on metabolic dysfunction-associated steatohepatitis: multicentre, double blind, randomised, placebo controlled trial. Bmj. 2025;389:e083735.
  - ○ Evidence regrading the effect of SGLT 2 inhibitors on histological endpoints in patients with MASH is rather limited. This is the first RCT suggesting that dapagliflozin can lead to MASH resolution and fibrosis improvement in people with MASH.
- Harrison SA, Bedossa P, Guy CD, Schattenberg JM, Loomba R, Taub R, et al. A Phase 3, Randomized, Controlled Trial of Resmetirom in NASH with Liver Fibrosis. N Engl J Med. 2024;390:497–509.
  - ○ Resmetirom was the first agent to receive regulatory approval for the management of non-cirrhotic MASH. This was bassed on the positive results of the MAESTRO trial where resmetirom improved both steatohepatitis and fibrosis in individuals wiith MASH.
